# Role of Chemokine Network in the Development and Progression of Ovarian Cancer: A Potential Novel Pharmacological Target

**DOI:** 10.1155/2010/426956

**Published:** 2009-12-14

**Authors:** Federica Barbieri, Adriana Bajetto, Tullio Florio

**Affiliations:** Laboratory of Pharmacology, Department of Oncology, Biology and Genetics, University of Genova, 16132 Genova, Italy

## Abstract

Ovarian cancer is the most common type of gynecologic malignancy. Despite advances in surgery and chemotherapy, the survival rate is still low since most ovarian cancers relapse and become drug-resistant. Chemokines are small chemoattractant peptides mainly involved in the immune responses. More recently, chemokines were also demonstrated to regulate extra-immunological functions. It was shown that the chemokine network plays crucial functions in the tumorigenesis in several tissues. In particular the imbalanced or aberrant expression of CXCL12 and its receptor CXCR4 strongly affects cancer cell proliferation, recruitment of immunosuppressive cells, neovascularization, and metastasization. In the last years, several molecules able to target CXCR4 or CXCL12 have been developed to interfere with tumor growth, including pharmacological inhibitors, antagonists, and specific antibodies. This chemokine ligand/receptor pair was also proposed to represent an innovative therapeutic target for the treatment of ovarian cancer. Thus, a thorough understanding of ovarian cancer biology, and how chemokines may control these different biological activities might lead to the development of more effective therapies. This paper will focus on the current biology of CXCL12/CXCR4 axis in the context of understanding their potential role in ovarian cancer development.

## 1. Introduction

Chemokines are small secreted cytokines, primarily involved in the regulation of the motility of hematopoietic cells (cells of the immune system) in their specific homing to lymphoid organs in normal hematopoiesis and during inflammation [1], through the activation of specific G-protein coupled receptors [2]. 

To date 53 human chemokines and 23 receptors have been cloned and characterized.

Chemokines display high structural homology and overlapping functions and often bind more than one receptor. In general, ligand binding causes chemokine receptor activation, hallmarked by the phosphorylation of C-terminal serine/threonine residues that, in turn, drives dissociation of heterotrimeric G-proteins into *α* and *βγ* subunits, inhibition of adenylyl cyclase activity, increased generation of inositol trisphosphate, intracellular calcium release, and the activation of phosphatidyl inositol 3 kinase (PI3K)/Akt cascade and Ras/MAP kinase signalling [3].

**Figure 1:**
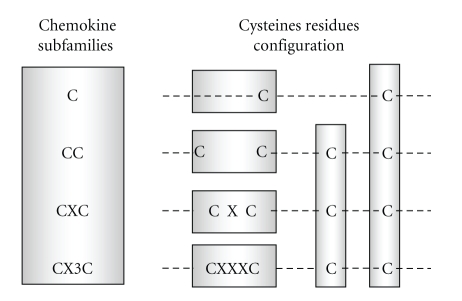
*Chemokine subfamilies classification. *The first cysteine (C) in the sequence forms a covalent bond with the third, the second and the fourth cysteines also form a disulfide bond to create the tertiary structure characteristic of chemokines. In the CC subfamily the first two cysteines are adjacent to each other, in the CXC group there is one amino acid between the first two cysteines, and in the CX3C group there are three amino acids between cysteines.

Chemokines are divided into subfamilies by structural and functional criteria. Structurally, chemokines are classified into four groups (C, CC, CXC, and CX3C) according to the number and location of the conserved cysteine residues in the primary structure of these molecules (Figure 1). The “C” group of chemokines (containing only two cysteines) consists of two molecules (XCL), namely, XCL1/lymphotactin and XCL2/SCM-1*β*, both binding the receptor XCR1. Lymphotactin, coded on human chromosome 1, attracts lymphocytes but not monocytes or neutrophils. 

Human “CC” chemokines (structurally characterized by four cysteines) includes 28 members, called CCL1-28 that bind at least 10 receptors (CCR1-10). CC chemokine targets include monocytes, T cells, dendritic cells, eosinophils, and basophils. Representative CC chemokines are CCL2 (also called monocyte chemotactic protein, MCP-1), CCL3 and CCL4 (macrophage inflammatory protein MIP-1*α* and MIP-1*β*), CCL5 (RANTES), and CCL11 (eotaxin). 

The “CXC” group (in which one amino acid is present between the first two cysteines) includes 21 ligands (CXCL1-21) mostly encoded on human chromosome 4. CXC chemokines bind at least 7 receptors (CXCR1-7) and mediate neutrophil chemotaxis. The CXC group can be divided into two main categories based on the presence of the tripeptide Glu-Leu-Arg (ELR) before the CXC motif (N-terminal domain). Representative CXC chemokines include CXCL8/IL-8, among the ELR-containing peptides and CXCL9/monokine-induced by IFN-*γ* (MIG), CXCL10/IFN-*γ* inducible protein-10 (IP-10), and CXCL12/stromal cell-derived factor-1 (SDF1) as ELR negative molecules. 

Lastly, the “CX3C” chemokines (three amino acids between the first two cysteines) are, to date, represented by a single peptide, namely, CX3CL1/fractalkine, which is encoded on human chromosome 16, binds the CX3CR1 receptor and regulates T cell trafficking and adhesion [4].

Functionally, chemokines, released upon inflammatory stimuli that induce leukocyte recruitment to damaged/infected sites, are considered as “inflammatory” [5] while chemokines that induce migration of leukocytes to lymphoid organs are considered “homeostatic” and are usually constitutively secreted by stromal cells expressed at these sites [6]. Homeostatic chemokines, such as CXCL12, coordinate cell trafficking and homing, which is essential during development and for homeostasis and function of the immune system. 

More recently, several extra-immunological functions were discovered for most of the components of the chemokine sub-families (for review see [7]). In particular, it was demonstrated that chemokines are major players during embryonic development, when their role as chemotactic mediators contribute to cell migration in the different body districts. Moreover, in the adult, chemokines play a relevant function in the central nervous system (CNS) where both ligands and receptors are expressed [8, 9]. At CNS level, chemokines control, among other functions, pain, alimentary behavior and glial responses to injuries [10–12].

## 2. Chemokines in Cancer

In cancer, genetic changes that accumulate in transformed cells are dependent on microenvironmental factors and control the development of the malignant process. In the past few years, a major role has been assigned to chemokines and their receptors as molecules that affect neoplastic development and progression.

Many chemokine/receptor pairs are expressed in tumors, not only by cancer cells but also by cells of the tumor microenvironment, including cells of the stroma (endothelial cells, fibroblasts) and leukocytes, thus contributing to the cross-talk between the tumor and its microenvironment to control tumor growth and progression [13].

In the malignancy context, chemokines play diverse effects, most of them deriving from their ability to induce cell migration. The ability of chemokines to enhance the motility of leukocytes, endothelial cells, and/or tumor cells is a key factor in determining the cancer establishment and progression. Depending on their specific expression pattern on target cells, on tumor type and on tumor microenvironment factors, several chemokines support malignancy, while others can at times inhibit this process [14].

The extensive research that was thus far performed on the roles of chemokines in cancer indicates that these molecules affect tumors mainly acting at four levels: (a) determine the extent and type of leukocyte infiltrates; (b) promote angiogenesis; (c) control site-specific metastasization; (d) affect tumor cells proliferation [15, 16].

The immune response induced by malignancy is clearly evident since many solid tumors are highly populated by host leukocytes that have migrated into the tumor from the systemic circulation. 

In tumors, leukocyte infiltrates may have either anti-cancer or cancer-promoting effects, depending on their type, their activity, and their modes of interaction with the tumor cells. In line with their classification as leukocyte chemoattractants, chemokines are released by tumor cells or by cells of their microenvironment and are able to induce the recruitment of different hematopoietic cell subtypes to tumors (T lymphocytes, macrophages, natural killer (NK) cells, neutrophils, eosinophils, and B cells). In particular, among CXC chemokines, CXCL9, CXCL10, and CXCL11 are induced by interferon *γ* (IFN*γ*) and are typical chemoattractants of NK cells [15]. Accordingly, overexpression of these chemokines by different experimental means leads to limitations in cancer development, associated with elevation in cytotoxic responses and with the creation of long-term antitumor immunity. These chemokines have additional antitumor activities but at the same time they may exert tumorigenic functions when acting directly on the tumor cells [17].

On the other hand, some chemokines may induce proangiogenic effects, leading to highly neovascularized tumors and increased metastatic spread. A large number of studies now clearly indicate that chemokines, mainly belonging to the CXC and CC subgroups, are important regulators of tumor angiogenesis. However, also on this parameter, opposite final effects may occur; some chemokines support the formation of new blood vessels, while others are angiostatic.

Angiogenic CXC chemokines promote the migration and proliferation of endothelial cells. Accordingly, they were shown to be potent tumor-supporting factors in a large variety of tumor types. For example, CXCL8 acts on endothelial cells mainly via their high affinity CXCR2 receptor [18]. Conversely, other CXC chemokines, including CXCL4, CXCL9, CXCL10, and CXCL11 are potent angiostatic factors. Their activity, via the CXCR3, receptor inhibits the neovascularization induced by powerful angiogenic factors. This activity of CXC chemokines, altogether with their ability to recruit antitumoral immune cells, led to the hypothesis of a function for these peptides as potential antitumoral factors. In contrast, CXCL12, another relevant member of this group, promotes tumor neoangiogenesis under specific conditions, thus being regarded as one of the most powerful promalignancy factors [19]. Many chemokines sustain cancer cell proliferation and survival, through an interaction with receptors expressed by the tumor cells. Such an activity was reported for CXCL8 that acts as an autocrine growth factor for human ovarian cancer Hey-A8 cell line [20]. Moreover, in vivo studies showed that CXCL8-overexpressing Hey-A8 cells are able to increase tumor cell growth, microvessel density, and the tumorigenic rate [20].

The chemokine CXCL12 also exerts a direct effect on tumor cell proliferation and survival in a high variety of tumors. In fact, altogether with its receptor CXCR4, CXCL12 constitutes the chemokine/receptor axis that attracted the greatest interest in oncology. This receptor-ligand system was reported to be overexpressed in several cancer types including acute and chronic leukemias [21] and solid tumors such as breast [22], colon [23], prostate [24], and ovarian cancers [25], glioblastomas [26, 27], melanomas [28], pituitary adenomas [29], and meningiomas [30, 31].

Physiologically, the CXCR4/CXCL12 axis is involved in migration of embryonic cells participating to the development of the central nervous system, bone marrow, and heart [32, 33]. Although for many years the interaction between CXCR4 and CXCL12 was thought to be unique, more recently, it was reported that CXCL12 binds also to another receptor, named CXCR7 [34]. CXCR7 is expressed in several cell types, including endothelial cells, T and B cells, dendritic cells, chondrocytes, endometrial stromal cells. The interaction of CXCL12 with CXCR4 mainly affects chemotaxis, while the binding to CXCR7 mediates proliferation in tumor cells [35]. Thus, CXCL12 can modulate the migration capacity of tumor cells and CXCR7 can enhance tumor growth.

**Figure 2:**
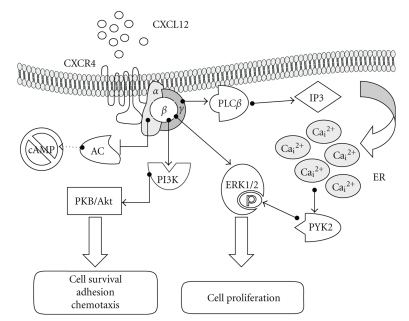
*Signaling pathways downstream CXCR4 receptor activation.* Upon CXCL12 binding the G protein complex dissociates into *α* and *βγ* subunits that trigger parallel signal transduction cascades culminating in tumor cell proliferation, migration, or survival. AC: adenylyl cyclase; ER: endoplasmic reticulum; PLC: phospholipase C; PI3K: phosphoinositide 3-kinase; PKB/Akt: protein kinase B; PKC: protein kinase C; PYK2: proline-rich tyrosine kinase 2; ERK1/2: extracellular regulated kinase.

Chemokines interacting to specific G protein coupled receptors (GPCRs) activate several signalling pathways in both normal and cancer cells. In normal cells, upon activation, CXCR4 dimerizes and transduces several intracellular signals (Figure 2). Most of them are PTX-sensitive and therefore dependent on activation of G_i_/G_o_ proteins, including G*α* and G*β γ*-mediated signals. G*α*_*ι*_ activation implies the inhibition of adenylyl cyclase (AC) function, thereby determining a decrease in the cytosolic concentration of cyclic AMP (cAMP), and leading to the inhibition of protein kinase A (PKA). G*α*_*q*_ transduces the CXCR4 signals through the activation of phospholipase C*β* (PLC) which increases inositol triphosphate and intracellular calcium levels. Chemokine signalling activated by G*βγ* induces a direct activation of PI3 kinase (PI3K), a survival regulator acting on effectors of apoptosis [36]. PI3K activation is often detected in cancer as mediator of the increased survival of the tumor cells. Importantly, beside survival, chemokines in general, and CXCL12 in particular, are also powerful activators of the MAP kinase (ERK1/2) cascade, the most studied proliferative mechanism responsible of tumor growth [30, 36]. Interestingly, ERK1/2 activation may involve either the classical Ras/Raf/MEK pathway or the activation of the cytosolic, Ca^++^-dependent tyrosine kinase Pyk2 [11]. However, in pituitary adenoma cell lines, CXCR4-induced activation of the calcium-dependent protein kinase Pyk2 was involved in a proliferative response independent by ERK1/2 and involving large conductance Ca^2+^-dependent K^+^ channels [37, 38]. Thus, the intracellular signaling involved in CXCL12 tumor cell proliferation is extremely dependent on the cell type analyzed.

 More recently, several studies proposed that CXCR4-dependent activation of ERK1/2 was mediated by transactivation of tyrosine kinase receptors. Cross-talk between growth factor and G protein-coupled receptors is now believed to play an important role in both normal and tumor responses. In particular, the transactivation of epidermal growth factor receptor (EGFR) and the activation of its downstream signaling pathways are critical for the mitogenic activity of different GPCR ligands, including chemokines. In the ovarian cancer cell line SKOV-3, CXCL8/IL-8 was shown to induce transient phosphorylation of EGFR and its association with the adaptor molecules Shc and Grb2, suggests an important cross-talk between chemokine and growth factor pathways [39]. In three other ovarian cancer cell lines (OC 314, OC 315, OC 316), it was demonstrated that CXCL12/CXCR4 interaction induces a dose-dependent cell proliferation through ERK1/2 and Akt activation that was dependent on EGFR phosphorylation caused by a mechanism involving the activity of the cytosolic tyrosine kinase c-Src [40]. Thus, a “cross-talk” between CXCL12/CXCR4 and EGFR intracellular pathways may link signals of cell migration and proliferation in ovarian cancer. A similar mechanism was also demonstrated in breast cancer cell lines, showing that estrogen-dependent proliferation involves the synthesis of CXCL12 (identified as estradiol-dependent gene), that causes an autocrine stimulation of CXCR4 and the subsequent activation of c-Src that, in turn, induces a ligand-independent activation of EGFR and ERK1/2 phosphorylation [41]. The transactivation of HER2/neu through CXCL12 stimulation of CXCR4 and Src activation cells was also demonstrated, in breast and prostate cancers [41–43].

## 3. Chemokines, Tumor-Specific Immune Responses, and Tumor Microenvironment

The interaction of chemokines, their receptors, growth factors, inflammatory with cancer cells forms a complex network at the tumor site, responsible for the overall progression or rejection of the tumor. In particular, chemokines play an essential role in coordinating the function of the immune system participating either in several steps of the antitumor immunity or in the regulation of the release of several mediators able to activate proangiogenic stimuli, and thus supporting tumor development.

**Figure 3:**
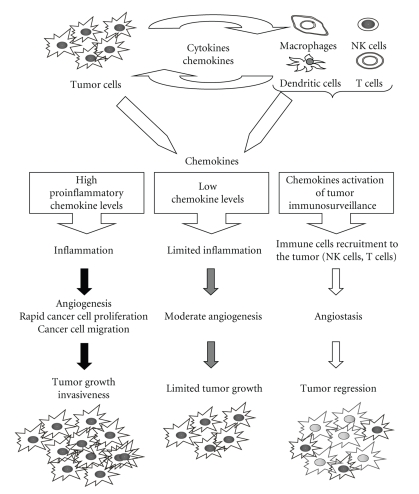
*Role of chemokines in the tumor-specific immune response. *The type and the amount of the chemokines secreted by tumor and inflammatory cells determine the extent and the effect of immune response leading to antitumor cytotoxic response, limited inflammatory, and vascular activation or potentiating tumor cell proliferation and neoangiogenesis.

 In fact, on one hand, chronic inflammation is often associated with cancer development [44] with an inflammatory component detectable also in the microenvironment of tumors non epidemiologically related to inflammation [45]. Moreover, several chemokines (mainly of the CXC/ELR+ family) are involved in regulation and recruitment of multiple cell types within tumor microenvironment, often possessing neoangiogenetic activity (Figure 3). Thus, chemokines may exert not only a direct effect on tumor cells but may control tumor growth also through the activation of their specific receptors expressed in a large number of stromal (fibroblasts and endothelial cells) and inflammatory cells. Importantly these cells also secrete a variety of chemokines, which regulate the migration of infiltrating macrophages, lymphocytes, dendritic cells, and neutrophils in response to a chemokine gradient [13]. 

 On the other hand, the activity of several chemokine may be detrimental for tumor growth causing the recruitment in tumor microenvironment cytotoxic T lymphocytes and NK cells responsible of the immunosurveillance against transformed cells (Figure 3).

 Among CXC-chemokines, CXCL12 is known to regulate the local immune response and is a potent chemoattractant for T cells, pre-B lymphocytes, and dendritic cells and its receptor CXCR4 is expressed by T lymphocytes, monocytes, neutrophils, and endothelial cells. This chemokine produced by different cell types in the tumor microenvironment modulates the activity of immunosuppressive cells (macrophages, neutrophils, T regulatory cells) contributing to tumor progression. 

The molecular pathways activated in tumor cells that control cancer-related immunity include transcription factors, such as nuclear factor-*κ*B (NF-*κ*B), hypoxia inducible factor *α* (HIF1*α*), and signal transducer and activator of transcription 3 (STAT3), which, in turn, control the production of other chemokines and inflammatory mediators (prostaglandins, cytokines). Altogether these factors trigger the recruitment of activated inflammatory cells generating the cancer-related inflammatory microenvironment. For example, tumor-associated leukocytes represent a source of growth factors, acting on tumor cells, and angiogenic factors. 

This dual role of chemokines in tumor development, to either eliminate malignant cells or escape the host immune control, was demonstrated in several tumor types [46]. For example, in melanoma, while chemokines contribute to the recruitment of CD8+ T lymphocytes expressing CXCR3 that infiltrate the tumor leading to the improvement of patient survival [22], the lack of critical chemokines (CCL2, CCL3, CCL4, CCL5, CXCL9, and CXCL10) in melanoma metastases may block the migration of activated T cells, which in turn could limit the effectiveness of antitumor immunity [47]. On the other hand, the aberrant expression of chemokines in tumors induces immunosuppression and favors tumor growth as shown in hepatocellular carcinoma, where high levels of CXCL9 and CXL10 have been associated with inhibition of CXCR3 expression by CD8+ T limphocytes, reduction of T-cell tumor infiltration and cytotoxic functions and tumor growth [48]. 

Another mechanism by which certain tumors evade the immune system is through the chemorepulsive activity of high levels of CXCL12. CXCL12 at low concentration (<10 nM) acts as a T cell chemoattractant [49], while higher concentrations of the chemokine can repel T cells in vitro and in vivo via a CXCR4 receptor-mediated mechanism [50]. 

In conclusion, according to which chemokines are released, completely opposite events may occur; abundant production of proinflammatory chemokines (i.e., ELR+ CXC-chemokines) can lead to a strong inflammatory response that potentiates angiogenesis, thus favoring a rapid neoplastic growth. Alternatively, high levels of monocytes and/or neutrophil infiltration, for example, in response to ELR- chemokines, can be associated with angiostasis, cytotoxicity, and possible tumor regression [19] (Figure 3). 

On the basis of these evidences, the characterization of the different chemokine networks in various types of cancer cells may foster better knowledge for understanding the immune-related mechanisms of cancer development and application in cancer immunotherapy.

## 4. Ovarian Cancer

Ovarian cancer causes more deaths than any other cancer of the female reproductive system, representing the world sixth most commonly diagnosed neoplasia among women [51]. Despite the high incidence and mortality rates, the etiology of this disease is poorly understood. Age and family history for the disease represent established risk factors for ovarian cancer; other possible risk factors include postmenopausal hormone-replacement therapy and lifestyle factors such as cigarette smoking and alcohol consumption. However, in many cases, the causes of ovarian cancer are yet to be identified. 

The progression of these tumors within the peritoneal cavity results in late diagnosis and high mortality rate. Indeed, the majority of patients are diagnosed with advanced disease and treated with surgery and postoperative cis-platinum- and taxane-based chemotherapy [52]. Moreover, several patients exhibit primary resistance to chemotherapy and approximately 70% achieve remission that is generally not durable, with an overall 5-year survival rate of 20–30% [53]. 

Ovarian cancers are histologically diverse: about 80% originates in the epithelium (epithelial ovarian cancer, EOC); the remaining 20% arise from other cell types (germ cell, sex cord-stromal, and mixed cell tumors) or are metastases to the ovary (most commonly, from the breast or gastro-intestinal tract tumors). EOC includes four histotypes (serous, mucinous, clear cell, and endometrioid) differing for epidemiologic, genetic changes, tumor markers, and response to therapy. The molecular events leading to the development of EOC are not yet clear, although if genetic and epigenetic alterations have commonly been observed. One of the most frequent genetic changes (about 70% of cases) is the mutation or loss of *TP53* function but, despite the great number of studies, its correlation with chemoresistance and prognostic impact are not yet fully proved [54]. Loss of heterozigosity and mutations *BRCA1* and *BRCA2* leading to inactivation of other genes, has been described in familial ovarian cancers [55]. Mutations, amplification, and overexpression of well-known oncogenes, such as *PI3K *subunit-*α*,* FGF1, MYC, EGFR, KRAS, HER2, *and* AKT2,* have been also associated with ovarian cancer [56].

 The different subtypes of ovarian cancer could be further subclassified according to tumor cell type grade, taking into account the different molecular characteristics: high grade serous cancers typically contain *BRCA1* and *TP53* mutations (up to 80%); low grade serous carcinomas often have mutations in the *KRAS *and* BRAF* genes (>60%), and low-grade endometrioid cancers are associated with mutations in the beta-catenin gene, *CTNNB1, PTEN, and PI3CA* and mucinous carcinomas frequently have mutations in *KRAS* and *TP53*. This subclassification of ovarian cancers is essential because different subtypes of ovarian cancer respond differently to treatment and have different prognoses [57].

 Some of the genes involved in ovarian cancer development control the activation of specific intracellular signalling pathways, particularly PI3K/Akt, EGFR, HER2/neu, PKC1, Src, and Ras, that are activated in more than half of ovarian neoplasms and thus could represent future targets for new anticancer agents. 

In particular, EGFR and HER2/neu activate signaling pathways (PI3K/Akt and ERK1/2 MAP kinase), leading to different cellular processes involved in tumor development, such as cell division and migration, adhesion, differentiation, and apoptosis [58]. Aberrant EGFR and HER2 expression was reported in ovarian carcinomas [59]. This evidence prompted the development of several strategies to target EGFR and HER2: monoclonal antibodies directed against the extracellular domain of the receptors or small molecules targeting the intracellular tyrosine kinase domains (tyrosine kinase inhibitors (TKIs)) are in various stages of clinical trials for ovarian cancer [60]. 

However, the molecular pathways underlying ovarian cancer progression are still poorly understood and currently the signaling pathway research identified promising novel candidates for cancer treatment and thus much effort has been made to establish signal transduction as target for therapy.

One of the major challenges for ovarian cancer clinical outcome is the occurrence of metastasis. Ovarian cancer spreads by direct seeding of cells into the peritoneal cavity where they form cancer nodules, by lymphatic dissemination to the pelvis, or, less frequently, by hematological diffusion to the parenchyma of the liver or lung. Ovarian cancer metastasis in the peritoneal cavity is not limited by anatomical barriers, thus peritoneal metastatic lesions can easily implant and give rise to ascitic tumor cells growing in plasma-derived exudate. Both tumor size and accumulation of ascites are inversely associated with survival [61].

Some of the EOC clinical features (failure to early disease detection, resistance to chemotherapy, high rate of recurrence) have been recently ascribed to the presence, as in other solid human cancer types, of cancer stem cells (CSCs) a rare cell subpopulation that maintain their tumorigenic potential after cytotoxic therapy. 

These tumor characteristics well fit with the known properties of CSC such as quiescence and elevated multidrug resistance activity, leading to insensitivity to cytotoxic drugs, and multipotency, resulting in diversity in histological phenotype associated with ovarian cancer. A large number of studies on tumor-initiating stem cells in hematological and solid cancers have been published (for review see [62]), although very few reports addressed the role of CSCs in EOC. Stem and progenitor-like cells able of self-renewal, pluripotency, differentiation in vitro and in vivo have been recently isolated from human epithelial ovarian cancers [63]. It was also hypothesized that putative ovarian CSC possesses an altered mitochondrial phenotype associated to its evolution towards tumorigenesis. Few studies showed the isolation of putative mouse ovarian CSC endowed with clonogenic, tumorigenic activity in vivo, and enhanced chemo-resistance in vitro [64, 65]. Another recent evidence, supporting the role of CSC in EOC, was reported by Alvero et al., describing that these cells have a distinctive genetic profile that confers them the capacity to form ascites and solid tumors, display chemoresistance, and promote tumor recurrence [66].

A further development of this research area is required to unequivocally define the contribution of CSC to human ovarian cancer development, and the signaling pathways involved. Importantly, these findings could lead to new therapeutic strategies to specifically target ovarian CSCs. Moreover, this knowledge is essential to understand the mechanisms underlying the risk factors for this important disease and is crucial for the development of effective screening protocols aimed at its early detection. On these bases, an improved understanding of the molecular biology of ovarian cancer may lead to the discovery of novel molecular targets for the treatment of ovarian cancer. 

## 5. Chemokines in Ovarian Cancer

One novel and, possibly, extremely relevant signaling pathway in ovarian cancer development, growth, and diffusion is represented by the chemokine system. In EOC there is now evidence for a complex chemokine network regulating autocrine/paracrine mechanisms, relevant for the biology of both normal and malignant ovarian cells [67, 68]. As outlined before, chemokines expressed in cells of tumor microenvironment can affect the type and the degree of the immune infiltrate in the tumor. Among chemokines, the CC subfamily, and particularly CCL2, is the most often expressed in ovarian cancer histotypes [69] being particularly involved in macrophage recruitment. Negus et al. [70] reported the expression of CCL2 and CCL5 in epithelial ovarian cancer cells and demonstrated the relationship between the presence of CCL2 with the extent of immune cell infiltration. More recently, the analysis of ovarian cancer ascitic fluid and ascite cells allowed the identification of the expression of CCL2,-3,-4,-5,-8, and -22, altogether with their receptors (namely, CCR1, -2a,-2b, -3,-4,-5, and-8), at mRNA and protein level [71]. However, a definite correlation between this expression pattern and the total cell counts in ascites or the stage of the disease still has not been completely reached. Indeed, tumors appear to utilize the same molecular mechanisms used by normal immune system to eliminate malignant cells. Concerning this topic, the influence of chemokines in the antitumor immune response has been described in a study that strongly supports the view that tumor-associated regulatory T cells (mediators of the immune tolerance by suppressing autoreactive T cells directed towards tumor antigens) impair the function of T effector cells in tumor-bearing patients [72]. Tumor tissue and ascites from patients with ovarian cancer contain high levels of cells with the hallmarks of regulatory T cells. These cells migrate into the tumor microenvironment in a process mediated by the chemokine CCL22 and are capable of suppressing antitumor responses. This specific recruitment of regulatory T cells represents a mechanism by which tumors may develop immune advantages and, as a consequence, the suggested inhibition of regulatory T cell migration or function using antibodies against CCL22, may represent a novel antitumor approach.

 One of the main features of all solid tumors is their dependence on neovascularization. Cancer cells recruit endothelial cells through the activity of several chemokines, cytokines, and growth factors. Angiogenesis is also critical for ascites development and metastasis in ovarian cancer. The role of chemokines in tumor angiogenesis is well known, and this process is mainly controlled by chemokines of the CXC family in a negative (angiostatic chemokines, ELR-) or positive manner (angiogenic, ELR+) [73]. In particular, CXCL8/IL-8 and CXCL1-3/GRO*α*, *β* and *γ*, and CXCL5/ENA-78 induce angiogenesis through the activation of direct mechanisms on endothelial cells [74]. In ovarian cancer, the existence of a direct relationship between the expression of angiogenic molecules and the pathological behavior of five different human ovarian cancers, xenografted in the peritoneal cavity of nude mice, was reported, demonstrating that the expression of CXCL8/IL-8 was associated with neovascularization and inversely correlated to survival [75].

Most chemokines sustain cancer cell proliferation and survival acting as autocrine factors, as reported for CXCL8 in Hey-A8 human ovarian cancer cells line blocked by specific neutralizing antibodies. Moreover, CXCL8-overexpressing cells when xenotransplanted in mice display an increased cell growth, microvessel density, and the tumorigenic rate [20]. Similarly, in vitro, IL-6 and CXCL8/IL-8 accelerate the proliferation rate of several EOC cell lines [76]. 

The metastatic spread of tumors is controlled by the microenvironment of the metastatic organ that supports the homing and the growth of the tumor cells. Several observations indicate that the tumor microenvironment at metastatic sites is enriched with chemokines and that tumor cells, expressing the cognate receptors, migrate and adhere in response to the chemokines promoting metastasis formation at these specific target organs.

In this respect, a very extensive research was performed on CXCL12/CXCR4 ligand/receptor pair in breast cancer, showing high expression of CXCL12 in target metastatic organs of breast tumor cells, and the expression of CXCR4 in tumor cells [77].

An important role for the CXCL12/CXCR4 axis in ovarian tumor metastasis was also identified and a correlation between the activity of this chemokine system and an enhanced intraperitoneal dissemination of EOC was described [78] (see below). However, it is clear that CXCL12 does not act by itself, and other pairs of chemokines of different families and their respective receptors are involved in metastasization. In EOC ascitic fluid and blood of patients the reduction of migration responses of monocyte/macrophages expressing several chemokine receptors (CCR1, CCR5, and CXCR4) produced in the tumor microenvironment, has been described [79]. In particular, the role of CCL11/eotaxin-1 in proliferation and invasion of ovarian cancer cells was analyzed. EOC overexpressed CCR2, 3, and 5, the cognate receptors of CCL11, with a strong positive correlation between tumor grade and the levels of each of these receptors. Interestingly, the inhibition of CXCL11 activity by neutralizing antibodies significantly increases cis-platinum response in ovarian carcinoma cells [80].

## 6. CXCL12/CXCR4 Expression in Ovarian Cancer and Its Role in Tumor Cell Proliferation and Metastases

In 2001 and 2002 Scotton et al. investigated, for the first time, the possible role of CXCL12 in ovarian cancer [25, 81]. In particular the expression of chemokines and their receptors was reported in ten primary ovarian tumors, six cell lines, and twenty ovarian cancer ascites. From these studies it was shown that CXCR4 was the only chemokine receptor expressed in these cells. Its expression was evidenced not only in tumor cells but also in mononuclear and endothelial cells within the tumor tissues; whereas endothelial cells in tissues adjacent to the tumors were negative. CXCR4 was also expressed in almost all the cells derived from ascites, and high concentrations of CXCL12 were detected in all ascitic fluids analyzed. 

Interestingly, CXCR4 and CXCL12 are not involved in the biology of normal ovarian epithelium since their expression was not detected in normal ovarian tissues and in healthy women with family history of ovarian cancer. In contrast, papillary serous and endometrioid ovarian tumors display a high expression of CXCL12. Low-grade ovarian cancers were reported to have a differential pattern of expression, with benign mucinous tumors negative but serous benign positive for the expression of this chemokine [25].

Subsequently, CXCR4 and CXCL12 expression in ovarian cancer have been confirmed by other studies [78, 82, 83].

More recent detailed analysis showed the expression of CXCR4 and CXCL12 also in normal ovary, but their localization was confined to the follicular cells and it was not detected in normal epithelium [82]. However, in consideration that 91% and 59% of ovarian cancers express CXCL12 and CXCR4, respectively, it was proposed that an overexpression of this chemokine system was present in ovarian malignant cells. In addition, the CXCR4/CXCL12 expression was associated with unfavorable prognosis with significantly reduced median disease progression-free survival [82]. Similarly, Kajiyama et al. [78] demonstrated that in patients whose tumors were positive for the expression of CXCR4, the overall survival was significantly worse than in patients negative for CXCR4 expression. Moreover, the level of CXCL12 in the ascites was directly related to the stages of disease. 

Thus, even if the published studies individually examined a small number of ovarian cancers, all these results agree that CXCL12/CXCR4 axis may be closely associated with the development of peritoneal metastasis and the prognosis of patients with epithelial ovarian carcinoma (EOC).

In vitro studies directly demonstrated that, in the presence of CXCL12, CXCR4 controls both ovarian cancer cell proliferation and migration, through the activation of the ERK1/2 and Akt pathway [40]. In addition, it was also demonstrated that CXCL12 effects on ovarian cancer cell lines are mediated by EGFR transactivation through a mechanism involving the activity of cytosolic tyrosine kinases, belonging to the c-Src family [84].

Inhibition of CXCR4 activity reduces intraperitoneal dissemination of ovarian cancer xenografts. In vitro, CXCL12 induces cell migration and invasion of the IGROV ovarian cancer cell line. Human peritoneal mesothelial cells (HPMCs), lining the peritoneal cavity, bind to EOC in the initial step of peritoneal metastasis. CXCL12 is predominantly expressed in HPMCs rather than in EOC cells, while CXCR4 was found in both EOC and HPMCs, thus creating an extracellular chemotactic milieu for EOC migration. Moreover, coculture experiments, using HPMCs and EOC, showed a strong increase in CXCL12 release, suggesting that some tumor-derived factors upregulate CXCL12 levels in ascites. TGF*β*1 may be one of these factors increasing CXCL12 production. AMD3100, a potent CXCR4 antagonist, reduced the formation of peritoneal metastasis in an in vivo experimental model, even if no significant differences in survival were observed between mice treated with AMD3100 and control mice [78]. Clinical trials should become feasible with the development of novel orally available CXCR4 inhibitors.

 Ovarian cancer metastasizes preferentially to local lymph nodes and peritoneum and, in contrast with breast cancer, only rarely in other organs such as liver, lung, and bones.

 Ovarian and breast cancers share the overexpression of HER2/neu, a member of the EGFR family. HER2/neu increases the metastatic potential in murine and human cell lines and induces mammary tumors and lung metastases in transgenic animal models [85]. CXCR4 plays an important role in targeting the metastasis of breast cancer. Malignant breast cancer cells express CXCR4, invade the extracellular matrix, and circulate in the blood and lymphatic vessels attracted by CXCL12 that is abundantly released by target metastatic organs. Recently, it has been demonstrated that the overexpression of HER2/neu in breast cancer enhances the metastatic potential through the upregulation of CXCR4, providing a link between CXCR4 and HER2/neu in tumor progression and metastasis [86]. 

 A similar study was performed in a cohort of 148 ovarian tumor samples by immunohistochemistry on tissue microarrays [87]. HER2/neu overexpression was found in a quarter of malignant tumors and associated with significantly shorter overall survival, in agreement with the majority of publications. However, HER2/neu positive patients did not show a higher expression of cytoplasmic CXCR4 staining, which was positive in over half of the cases and closely correlated with CXCL12 expression [87]. The lack of influence of CXCR4 in ovarian cancer could reflect the peculiar characteristics of the metastatic process in ovarian cancer as compared to other cancers such as colon [88], non-small-cell lung cancer [89], gliomas [90], malignant melanomas [28], oral squamous carcinoma [91], and adult acute myeloid leukemia [92]. In these types of cancer, distant metastasis, favored by CXCL12 expression on target organs, significantly influences the survival, while in ovarian cancer the recurrence in the pelvis and in the peritoneum is the main cause for death. Nevertheless, it needs to take in account that, in this study, CXCR4 and HER2/neu expression was evaluated in paraffin-embedded tissues blocks and their histology and grading was related with survival of patients, without analysis of the metastatic sites. 

 Thus, the presence of high level of CXCL12 in the ascitic fluids as well as the inhibition of intraperitoneal dissemination of ovarian cancer xenografts by CXCR4 antagonist suggests that CXCR4/CXCL12 axis may be important in the invasion of ovarian cancer cells and further studies will be necessary to better deepen this important question. 

The published studies dealing with the expression of CXCL12 and CXCR4 in human ovarian cancer cell lines, tumor biopsies and ascite cells are summarized in Tables 1 and 2, respectively.

Interestingly, also in other gynecological cancers, such as cervical and endometrial carcinomas, chemokines were reported to have a pathogenic role. 

Most of the early studies on the role of CXCR4 in cervical cancer were performed using the HeLa cell line (cervical adenocarcinoma-derived cell), the first immortalized cell line developed for research purposes [95, 96]. CXCL12 stimulation of HeLa cells induces increase of intracellular calcium concentrations, activation of ERK1/2 MAP kinase, PI3K-Akt and Jak-STAT pathways; all these signals cooperate in cell migration and spreading. Studies on human cervical carcinoma (HCC) demonstrated high expression of CXCR4 in HCC-derived cell lines and in tissue sections, while normal cervical epithelium was negative [97]. CXCL12 binding to CXCR4 induces cell movement, cytoskeleton reorganization, and gene activation, synergizing with hepatocyte growth factor. In fact, metastasization of cervical adenocarcinoma or squamous cell carcinomas is more frequent in tumors expressing high levels of CXCR4 than tumors that express either low levels or are negative for CXCR4 [98, 99]. Also CCR7 levels in cervical adenocarcinoma/squamous cells are associated with invasion of lymph nodes as well as tumor cell proliferation and survival. CXCR4 and CCR7 expression is significantly higher in patients with larger tumor size, deep stromal invasion, lymph-vascular space involvement, or lymph node metastasis [100]. As far as endometrial cancer, studies on CXCR4 and CXCL12 expression revealed that CXCR4 is overexpressed in endometrial cancers as compared with normal tissues, whereas CXCL12 was overexpressed in normal mucosa. In addition, in vivo cell migration may be contrasted by CXCR4 neutralizing monoclonal antibody that reduces size and number of the metastasis in all target organs (peritoneum, lung, liver) [101]. However, a different study shows that CXCR4 expression was inversely related to tumor grade and patient outcome [100]. On the contrary, no difference between cancer and normal tissues was reported as far as CXCR7 expression [101].

## 7. CXCR4/CXCL12 and Primordial Germ Cells Development

Ovarian germ cell tumors (OGCTs) account for about 20% of all ovarian neoplasms and constitute the second largest group of ovarian cancer mainly affecting young women (58% of all ovarian tumors in women younger than the age 20 years). They histogenetically derive from primordial germ cells and differ with regard to clinical presentation, tumor biology, and histology. OGCTs include both benign (predominantly) and malignant (MOGCT) subtypes. MOGCTs are rare but aggressive and very curable tumors, accounting for approximately 1-2% of all ovarian malignancies [102]. 

 In most organisms primordial germ cells (PGCs) migrate through developing embryo to reach the location where the gonad develop. In this process somatic cells of the gonads support the proper development of germ cells, the lineage that gives rise to sperm and eggs. 

 In mice lacking CXCL12, the colonization of gonads by PGCs is impaired [103]. CXCR4 is expressed in mouse germ cells; whereas expression of its ligand is high in the genital, the target of the migrating cells. In fact, CXCL12 is essential for homing of PGCs into genital ridges but is not required for direct migration through tissues of embryos [104, 105]. Different studies demonstrated that in mouse germ cell migration and survival requires the CXCL12/CXCR4 interaction [106, 107]. 

Recently, it was reported that also the other CXCL12 receptor, CXCR7, takes an active part in the migration of PGCs; knockdown of CXCR7 gene results in impaired polarity and aberrant migration of PGCs [108]. Unlike CXCR4, CXCR7 function was found to be required in tissues surrounding the migrating cells (where it is found primarily in intracellular structures) rather than in PGCs. It was suggested that the key role of CXCR7 is to bind and internalize CXCL12 thereby controlling the level of the diffusible chemokine in the extracellular space [108]. Thus, CXCR7 may act as a high-affinity decoy receptor to facilitate the migration of PGCs by shaping the distribution of the chemokine in the environment [109].

CXCR4 and CXCL12 control cell migration in several normal and pathological conditions [103–105, 108, 109]. In neonatal mice CXCL12/CXCR4 signalling contributes to maintain the size and longevity of the primordial follicle pool [106]. CXCL12/SDF1*α* and *β* (but not CXCL12/SDF1*γ*) transcript variants were identified in cultured neonatal and adult ovary by microarray analysis and RT-PCR. In neonatal tissues both CXCL12 and CXCR4 display similar expression pattern. They are detected in primordial and primary/secondary oocytes with lower level of staining in the interstitial tissues and granulosa cells. The primordial oocytes are essentially “resting” cells with limited metabolic capability suggesting that the presence of CXCR4/CXCL12 may be with index of an essential role for this chemokin system within the follicle [106].

Recently it was proposed an innovative hypothesis concerning the cell type by which ovarian epithelial tumors may rise [110]. In fact, it was widely accepted that the origin of epithelial ovarian tumors derives from the mesothelial cell layer lining the ovary surface (ovarian coelomic epithelium). However, it was observed that ovarian epithelial neoplasms are remarkably similar to epithelial cells from extra-ovarian sites in the female reproductive tract. The three most common subtypes of these tumors, referred as serous, endometrioid, and mucinous are morphologically identical to carcinomas of the fallopian tube, endometrium, and endocervix, respectively. It has been suggested that ovarian epithelial cells could arise from tissues that are embryological derived from the Mullerian ducts. 

In other types of cancer the physiological role of CXCR4/CXCL12, carried out during the embryonic development, is turned in the ability to influence cell migration and spreading in cancer. Thus, studying CXCR4/CXCL12 function in epithelial ovarian cancer, the choice of an incorrect cell controls could bring wrong conclusions. It could be possible that a deregulated CXCR4/CXCR7/CXCL12 axis could be already evident in early stage of illness.

## 8. CXCR4 as a Potential Therapeutic Target

All the data previously discussed provide the rationale for targeting CXCR4 in cancer. In particular, this notion is further supported by the different mechanisms resulting from CXCR4 inhibition: (a) the reversal of stromal cell interactions responsible of tumor cell survival; (b) the blockade of proangiogenic activity of CXCL12 and the reduction of the dissemination and migration ability of tumor cells; (c) the blockade of tumor growth through autocrine/paracrine signaling mediated by CXCL12/CXCR4 interaction; (d) the mobilization of tumor cells from tissues to increase their sensitivity to conventional chemoterapeutic agents.

 On these premises, several molecules able to antagonize CXCR4 activity in response to CXCL12 have been identified. To date four types of CXCR4 inhibitors have been described: (1) peptide-based antagonists, (2) nonpeptidic antagonists; (3) neutralizing antibodies for CXCR4; (4) modified CXCL12 peptides, endowed of antagonistic activity.

The first small peptide antagonists of CXCR4 (named T22, T134, and T140) [111, 112] were discovered screening compounds with potential anti-HIV-1 activity (CXCR4 acts also as coreceptor in T-cell line tropic HIV infection [95, 96]). In particular T22 blocks CXCR4 activity by binding the receptor region involved in HIV-1 entry into the cell. T140, the most active antagonist [113], is a 14-residues peptide with the main limitation in the low stability in serum. Thus, this shortcome was overcome by modified structural analogs (T14003 and TC14012) [114]. Recently, T140 synthesize as cyclic peptide (FC131) [115], and a new antagonist (named POL3026) [116], with better pharmacokinetic properties and potent CXCR4 antagonist activity, have been described. In cancer, T140 efficacy to block CXCR4 has been reported in different tumor models in vivo and in vitro, including leukemia [117], breast [118] and lung cancers [119], and malignant melanoma [120]. T140 and TN14003 are currently in clinical development for B-cell homing [117]. 

Among nonpeptidic small molecules, cyclams and bicyclams such as AMD3100 are endowed of CXCR4 antagonistic properties [121] and weak partial agonist activity [122–124].

Antitumor efficacy of AMD3100 was demonstrated in breast cancer where it blocks CXCL12-induced HER2/neu activation in vitro [125] and inhibits tumor growth in vivo [126]. Its antineoplastic activity has been also demonstrated in pancreatic cancer cells, colorectal and glioblastoma tumor xenografts [127–129]. Moreover, playing CXCR4 a key role in cross-talk between leukemia cells and their microenvironment, the potential use of this drug in hematological cancer has been widely studied and it is currently used in clinics for the mobilization of hematopoietic stem cells [130, 131].

Indeed, in hematological malignancies, tumor cells use CXCR4 for dissemination and progression of the disease, because interactions of CXCR4 with its ligand are critical for hematopoietic cells trafficking and homing to lymphatic tissues [132]. Stromal cells within bone marrow microenvironment constitutively secrete CXCL12 and the activation of CXCR4 induces leukemia cell migration to the marrow microenvironment, providing growth and drug resistance signals. AMD3100 is able to mobilize leukemia cells from their stromal microenvironment and inhibits adhesive tumor-stroma interactions, thus making leukemia cells accessible to conventional drugs [133]. 

AMD3100 has also been used as agent that disrupts interaction with the bone marrow microenvironment in multiple myeloma cells [134] and in between mantle cell lymphoma cells [135]. Therefore, targeting the CXCR4/CXCL12 axis is attractive therapeutic approach in leukemia patients. It was shown that in leukemia cells, several growth and survival factors from the tumor microenvironment, including CXCR4 activation, induce PI3K activation. Therefore, the activity of isoform-selective PI3K inhibitors was investigated to indirectly block CXCL12/CXCR4 signals in chronic cell leukemia leading migration, stromal cell interactions, and stromal cell-mediated drug resistance [136]. 

AMD3100 was successfully used in both in vitro and in vivo experiments carried out in ovarian cancer cells [25].

Antibodies against CXCR4 have been reported to affect HIV-1 infection and cancer cell migration [137]. The limiting point for monoclonal antibody therapeutic development is due to the high frequency of heterogeneous conformation of CXCR4 and posttranslational modification that reduce the antibody specificity and function [138].

Antichemokine activity was also identified in some natural compounds. Soybean and cruciferous vegetables have been implicated in the protection against spontaneous and carcinogen-induced cancers although the mechanisms for this anticarcinogenicity are not fully elucidated. Epidemiologic studies in Asian women indicate that consumption of a traditional diet high in soy confers significant protection against breast cancer [139].

3,3′-Diindolylmethane (DIM) and genistein, dietary phytoestrogens, belonging to isoflavone class of flavonoids, have anticarcinogenic activities [140]. Recently, Hsu et al. demonstrated that DIM inhibits the chemotactic and invasive potential of breast and ovarian cancer cells especially through an estrogen-independent mechanism, reducing the chemotaxis towards CXCL12. In addition, downregulation of CXCR4 and CXCL12 and inhibition of chemotaxis and chemoinvasion in breast and ovarian cancer cells toward CXCL12 are among of the biological effects of genistein [93] likely through the inhibition of the estrogen dependent CXCL12 mRNA synthesis.

## 9. Conclusions

One of the topics emerging from this review is that ovarian cancer growth and metastasis can be controlled by immunomodulatory and chemotactic chemokines. Indeed, chemokine/chemokine receptor systems attract increasing attention as anticancer strategies due to their direct involvement in almost every aspect of tumorigenesis. Thus, further biology and pharmacology studies have to be developed to fully address the chemokinergic system as an ideal target for the inhibition of tumor proliferation, angiogenesis, invasion, and metastasization.

## Figures and Tables

**Table 1:** CXCR4 and CXCL12 expression in human ovarian cancer cell lines.

Ovarian cancer cell lines	CXCR4 expression		CXCL12 expression		References
	Protein	mRNA	Protein	mRNA	

IGROV, CAOV-3, PEO1, PEO14	X	X	X	X	[81]
OVCAR-3, SKOV-3	Not detected	Not detected	Not detected	Not detected	[81]
IGROV, CAOV-3			X	X	[25]
OC 314, OC 315, OC 316	X	X		X	[40]
SKOV-3, RMG-I, NOS-2, KOC-7C,	X		X	X	[78]
ES2, NOS-4	X				[78]
BG-1	X	X	X	X	[93, 94]

**Table 2:** CXCR4 and CXCL12 expression in human ovarian carcinomas and tumor ascite cells.

Ovarian carcinoma	CXCR4 expression		CXCL12 expression		References
Solid tumors (positive/total, %)	Protein	mRNA	Protein	mRNA	

8/10, 80%	X	X	X	X	[81]
19/20, 95%		X		X	[81]
16/18, 88%	X		X		[25]
26/44, 60%	X				[82]
40/44, 91%			X		[82]
27/30 (metastatic), 91%			X		[82]
23/30 (metastatic), 77%	X				[82]
12/12, 100%	X	X	X	X	[83]
119/128, 93%	X				[87]
128/128, 100%			X		[87]
16/36, 44%	X				[78]

Ascites (positive/total, %)					
63/63, 100%			X		[81]
26/26, 100%			X		[78]
